# Supplementary material for: A new hypothesis for the origin of Amazonian Dark Earths
Source: Nat Commun. 2021 Jan 4;12:127. doi: 10.1038/s41467-020-20184-2 (PMC7782733; doi:10.1038/s41467-020-20184-2)
Supplement: Supplementary file 1 — Supplementary Information [file 41467_2020_20184_MOESM1_ESM.pdf]

**Supplementary Information** for A NEW HYPOTHESIS FOR THE ORIGIN OF AMAZONIAN DARK EARTHS by SILVA et al.

**Table S1.** Correlations between the total concentration of 28 elements measured in all maximum contrast ADE and Ultisol soil samples.

| | Mg | P | K | Ca | Ti | Li | Be | Al | V | Cr | Mn | Fe | Co | Ni |
|---|---|---|---|---|---|---|---|---|---|---|---|---|---|---|
| Mg | 1.0000 | 0.8102 | 0.9275 | 0.8925 | -0.5655 | 0.9128 | 0.7412 | -0.3370 | -0.2011 | -0.1635 | 0.9315 | -0.2783 | 0.9285 | 0.7538 |
| P | 0.8102 | 1.0000 | 0.8179 | 0.8063 | -0.5348 | 0.8981 | 0.8302 | -0.0555 | 0.1312 | 0.1343 | 0.7175 | 0.0391 | 0.8156 | 0.7654 |
| K | 0.9275 | 0.8179 | 1.0000 | 0.8357 | -0.4962 | 0.9356 | 0.7682 | -0.2856 | -0.1659 | -0.1231 | 0.8736 | -0.1931 | 0.9260 | 0.7163 |
| Ca | 0.8925 | 0.8063 | 0.8357 | 1.0000 | -0.5821 | 0.8345 | 0.6699 | -0.3561 | -0.2236 | -0.2050 | 0.8560 | -0.2685 | 0.8228 | 0.6851 |
| Ti | -0.5655 | -0.5348 | -0.4962 | -0.5821 | 1.0000 | -0.5251 | -0.3481 | 0.5229 | 0.3415 | 0.3754 | -0.5532 | 0.5234 | -0.4521 | -0.4653 |
| Li | 0.9128 | 0.8981 | 0.9356 | 0.8345 | -0.5251 | 1.0000 | 0.8604 | -0.1729 | -0.0085 | 0.0252 | 0.8528 | -0.1258 | 0.9403 | 0.7882 |
| Be | 0.7412 | 0.8302 | 0.7682 | 0.6699 | -0.3481 | 0.8604 | 1.0000 | 0.2557 | 0.4206 | 0.4420 | 0.6281 | 0.2554 | 0.8287 | 0.7705 |
| Al | -0.3370 | -0.0555 | -0.2856 | -0.3561 | 0.5229 | -0.1729 | 0.2557 | 1.0000 | 0.9329 | 0.9054 | -0.4792 | 0.9212 | -0.2003 | -0.0451 |
| V | -0.2011 | 0.1312 | -0.1659 | -0.2236 | 0.3415 | -0.0085 | 0.4206 | 0.9329 | 1.0000 | 0.9645 | -0.3452 | 0.8808 | -0.0602 | 0.1128 |
| Cr | -0.1635 | 0.1343 | -0.1231 | -0.2050 | 0.3754 | 0.0252 | 0.4420 | 0.9054 | 0.9645 | 1.0000 | -0.2771 | 0.8376 | -0.0000 | 0.1349 |
| Mn | 0.9315 | 0.7175 | 0.8736 | 0.8560 | -0.5532 | 0.8528 | 0.6281 | -0.4792 | -0.3452 | -0.2771 | 1.0000 | -0.4221 | 0.9125 | 0.6819 |
| Fe | -0.2783 | 0.0391 | -0.1931 | -0.2685 | 0.5234 | -0.1258 | 0.2554 | 0.9212 | 0.8808 | 0.8376 | -0.4221 | 1.0000 | -0.1779 | -0.0207 |
| Co | 0.9285 | 0.8156 | 0.9260 | 0.8228 | -0.4521 | 0.9403 | 0.8287 | -0.2003 | -0.0602 | -0.0000 | 0.9125 | -0.1779 | 1.0000 | 0.7670 |
| Ni | 0.7538 | 0.7654 | 0.7163 | 0.6851 | -0.4653 | 0.7882 | 0.7705 | -0.0451 | 0.1128 | 0.1349 | 0.6819 | -0.0207 | 0.7670 | 1.0000 |
| Cu | 0.9272 | 0.8657 | 0.9361 | 0.8751 | -0.5279 | 0.9236 | 0.7298 | -0.3123 | -0.1874 | -0.1534 | 0.9120 | -0.2075 | 0.9156 | 0.7560 |
| Zn | 0.9309 | 0.8374 | 0.9167 | 0.9013 | -0.5149 | 0.9049 | 0.7109 | -0.3282 | -0.2134 | -0.1790 | 0.9103 | -0.2270 | 0.9062 | 0.7499 |
| As | -0.1708 | 0.1645 | -0.1612 | -0.2006 | 0.2207 | 0.0000 | 0.4272 | 0.8744 | 0.9658 | 0.9263 | -0.3095 | 0.8232 | -0.0466 | 0.1386 |
| Se | -0.5374 | -0.4456 | -0.5565 | -0.5093 | 0.4850 | -0.4581 | -0.0675 | 0.6867 | 0.6463 | 0.6436 | -0.5545 | 0.5408 | -0.4017 | -0.2949 |
| Rb | 0.9298 | 0.8119 | 0.9607 | 0.8414 | -0.4990 | 0.8837 | 0.6705 | -0.3734 | -0.2564 | -0.2111 | 0.9026 | -0.2537 | 0.8984 | 0.6873 |
| Sr | 0.8257 | 0.8883 | 0.7985 | 0.8389 | -0.4319 | 0.8556 | 0.8362 | 0.0662 | 0.1848 | 0.1750 | 0.7066 | 0.1508 | 0.8029 | 0.7276 |
| Mo | -0.1787 | 0.1661 | -0.1297 | -0.1609 | 0.4302 | -0.0230 | 0.2946 | 0.8164 | 0.8139 | 0.7978 | -0.3226 | 0.8440 | -0.0827 | 0.0756 |
| Ag | 0.0984 | 0.1413 | 0.0642 | 0.0525 | -0.0442 | 0.1365 | 0.3603 | 0.3539 | 0.3840 | 0.3978 | 0.0440 | 0.2902 | 0.1491 | 0.1722 |
| Cd | 0.9037 | 0.8402 | 0.8977 | 0.9419 | -0.5354 | 0.8879 | 0.7050 | -0.3443 | -0.2253 | -0.1980 | 0.9020 | -0.2433 | 0.8888 | 0.7225 |
| Cs | 0.8786 | 0.7559 | 0.9091 | 0.8034 | -0.4512 | 0.8184 | 0.5884 | -0.3888 | -0.3069 | -0.2770 | 0.8656 | -0.2589 | 0.8410 | 0.6374 |
| Ba | 0.9289 | 0.8593 | 0.9120 | 0.9074 | -0.5350 | 0.8937 | 0.7411 | -0.2748 | -0.1533 | -0.1322 | 0.8869 | -0.1643 | 0.8993 | 0.7518 |
| Tl | 0.9565 | 0.8592 | 0.9350 | 0.8945 | -0.5766 | 0.9346 | 0.7758 | -0.3263 | -0.1623 | -0.1113 | 0.9462 | -0.2485 | 0.9351 | 0.7597 |
| Pb | 0.1711 | 0.4208 | 0.1512 | 0.1391 | -0.0907 | 0.3368 | 0.7051 | 0.6686 | 0.8232 | 0.8108 | 0.0512 | 0.5738 | 0.3002 | 0.4064 |
| U | 0.0848 | 0.1872 | 0.0230 | -0.0262 | 0.0610 | 0.1948 | 0.5250 | 0.6057 | 0.7226 | 0.7576 | 0.0201 | 0.4406 | 0.2265 | 0.2709 |

| | Cu | Zn | As | Se | Rb | Sr | Mo | Ag | Cd | Cs | Ba | Tl | Pb | U |
|---|---|---|---|---|---|---|---|---|---|---|---|---|---|---|
| Mg | 0.9272 | 0.9309 | -0.1708 | -0.5374 | 0.9298 | 0.8257 | -0.1787 | 0.0984 | 0.9037 | 0.8786 | 0.9289 | 0.9565 | 0.1711 | 0.0848 |
| P | 0.8657 | 0.8374 | 0.1645 | -0.4456 | 0.8119 | 0.8883 | 0.1661 | 0.1413 | 0.8402 | 0.7559 | 0.8593 | 0.8592 | 0.4208 | 0.1872 |
| K | 0.9361 | 0.9167 | -0.1612 | -0.5565 | 0.9607 | 0.7985 | -0.1297 | 0.0642 | 0.8977 | 0.9091 | 0.9120 | 0.9350 | 0.1512 | 0.0230 |
| Ca | 0.8751 | 0.9013 | -0.2006 | -0.5093 | 0.8414 | 0.8389 | -0.1609 | 0.0525 | 0.9419 | 0.8034 | 0.9074 | 0.8945 | 0.1391 | -0.0262 |
| Ti | -0.5279 | -0.5149 | 0.2207 | 0.4850 | -0.4990 | -0.4319 | 0.4302 | -0.0442 | -0.5354 | -0.4512 | -0.5350 | -0.5766 | -0.0907 | 0.0610 |
| Li | 0.9236 | 0.9049 | 0.0000 | -0.4581 | 0.8837 | 0.8556 | -0.0230 | 0.1365 | 0.8879 | 0.8184 | 0.8937 | 0.9346 | 0.3368 | 0.1948 |
| Be | 0.7298 | 0.7109 | 0.4272 | -0.0675 | 0.6705 | 0.8362 | 0.2946 | 0.3603 | 0.7050 | 0.5884 | 0.7411 | 0.7758 | 0.7051 | 0.5250 |
| Al | -0.3123 | -0.3282 | 0.8744 | 0.6867 | -0.3734 | 0.0662 | 0.8164 | 0.3539 | -0.3443 | -0.3888 | -0.2748 | -0.3263 | 0.6686 | 0.6057 |
| V | -0.1874 | -0.2134 | 0.9658 | 0.6463 | -0.2564 | 0.1848 | 0.8139 | 0.3840 | -0.2253 | -0.3069 | -0.1533 | -0.1623 | 0.8232 | 0.7226 |
| Cr | -0.1534 | -0.1790 | 0.9263 | 0.6436 | -0.2111 | 0.1750 | 0.7978 | 0.3978 | -0.1980 | -0.2770 | -0.1322 | -0.1113 | 0.8108 | 0.7576 |
| Mn | 0.9120 | 0.9103 | -0.3095 | -0.5545 | 0.9026 | 0.7066 | -0.3226 | 0.0440 | 0.9020 | 0.8656 | 0.8869 | 0.9462 | 0.0512 | 0.0201 |
| Fe | -0.2075 | -0.2270 | 0.8232 | 0.5408 | -0.2537 | 0.1508 | 0.8440 | 0.2902 | -0.2433 | -0.2589 | -0.1643 | -0.2485 | 0.5738 | 0.4406 |
| Co | 0.9156 | 0.9062 | -0.0466 | -0.4017 | 0.8984 | 0.8029 | -0.0827 | 0.1491 | 0.8888 | 0.8410 | 0.8993 | 0.9351 | 0.3002 | 0.2265 |
| Ni | 0.7560 | 0.7499 | 0.1386 | -0.2949 | 0.6873 | 0.7276 | 0.0756 | 0.1722 | 0.7225 | 0.6374 | 0.7518 | 0.7597 | 0.4064 | 0.2709 |
| Cu | 1.0000 | 0.9669 | -0.1677 | -0.6050 | 0.9577 | 0.8372 | -0.1143 | 0.0309 | 0.9523 | 0.9413 | 0.9667 | 0.9488 | 0.1251 | -0.0075 |
| Zn | 0.9669 | 1.0000 | -0.1954 | -0.5917 | 0.9337 | 0.8339 | -0.1296 | 0.0171 | 0.9568 | 0.9111 | 0.9571 | 0.9364 | 0.1077 | -0.0343 |
| As | -0.1677 | -0.1954 | 1.0000 | 0.6293 | -0.2335 | 0.1994 | 0.7618 | 0.4192 | -0.2098 | -0.2927 | -0.1304 | -0.1309 | 0.8584 | 0.7494 |
| Se | -0.6050 | -0.5917 | 0.6293 | 1.0000 | -0.6478 | -0.3214 | 0.4171 | 0.4014 | -0.5620 | -0.6737 | -0.5531 | -0.5375 | 0.5209 | 0.6198 |
| Rb | 0.9577 | 0.9337 | -0.2335 | -0.6478 | 1.0000 | 0.7805 | -0.1630 | -0.0170 | 0.9102 | 0.9661 | 0.9327 | 0.9411 | 0.0367 | -0.0671 |
| Sr | 0.8372 | 0.8339 | 0.1994 | -0.3214 | 0.7805 | 1.0000 | 0.1977 | 0.2046 | 0.8284 | 0.7511 | 0.8833 | 0.8195 | 0.4513 | 0.2449 |
| Mo | -0.1143 | -0.1296 | 0.7618 | 0.4171 | -0.1630 | 0.1977 | 1.0000 | 0.2110 | -0.1477 | -0.1703 | -0.0922 | -0.1386 | 0.5583 | 0.4266 |
| Ag | 0.0309 | 0.0171 | 0.4192 | 0.4014 | -0.0170 | 0.2046 | 0.2110 | 1.0000 | 0.0354 | -0.0841 | 0.0498 | 0.1123 | 0.4981 | 0.5043 |
| Cd | 0.9523 | 0.9568 | -0.2098 | -0.5620 | 0.9102 | 0.8284 | -0.1477 | 0.0354 | 1.0000 | 0.8842 | 0.9533 | 0.9329 | 0.1166 | -0.0391 |
| Cs | 0.9413 | 0.9111 | -0.2927 | -0.6737 | 0.9661 | 0.7511 | -0.1703 | -0.0841 | 0.8842 | 1.0000 | 0.9229 | 0.8757 | -0.0531 | -0.1431 |
| Ba | 0.9667 | 0.9571 | -0.1304 | -0.5531 | 0.9327 | 0.8833 | -0.0922 | 0.0498 | 0.9533 | 0.9229 | 1.0000 | 0.9306 | 0.1677 | 0.0051 |
| Tl | 0.9488 | 0.9364 | -0.1309 | -0.5375 | 0.9411 | 0.8195 | -0.1386 | 0.1123 | 0.9329 | 0.8757 | 0.9306 | 1.0000 | 0.2105 | 0.0871 |
| Pb | 0.1251 | 0.1077 | 0.8584 | 0.5209 | 0.0367 | 0.4513 | 0.5583 | 0.4981 | 0.1166 | -0.0531 | 0.1677 | 0.2105 | 1.0000 | 0.8519 |
| U | -0.0075 | -0.0343 | 0.7494 | 0.6198 | -0.0671 | 0.2449 | 0.4266 | 0.5043 | -0.0391 | -0.1431 | 0.0051 | 0.0871 | 0.8519 | 1.0000 |

**Supplementary Figure 1. (A)** Principal component analysis of total concentrations (28 elements) ordinated by soil type (Ultisol = triangles and ADE = circles), clustered primarily along principal component 1 (X axis) and depth (red = 100 cm and yellow = 10 cm), clustered primarily along principal component 2 (Y axis). **(B)** Contribution of each element toward separation along the top three ranked principal components (PC 1, 2, 3).

**A**

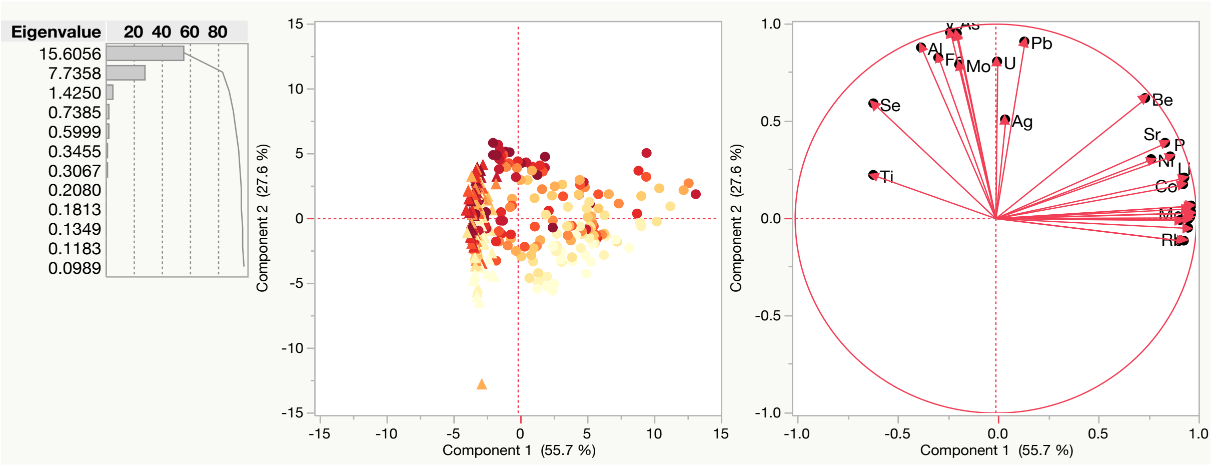


**B**

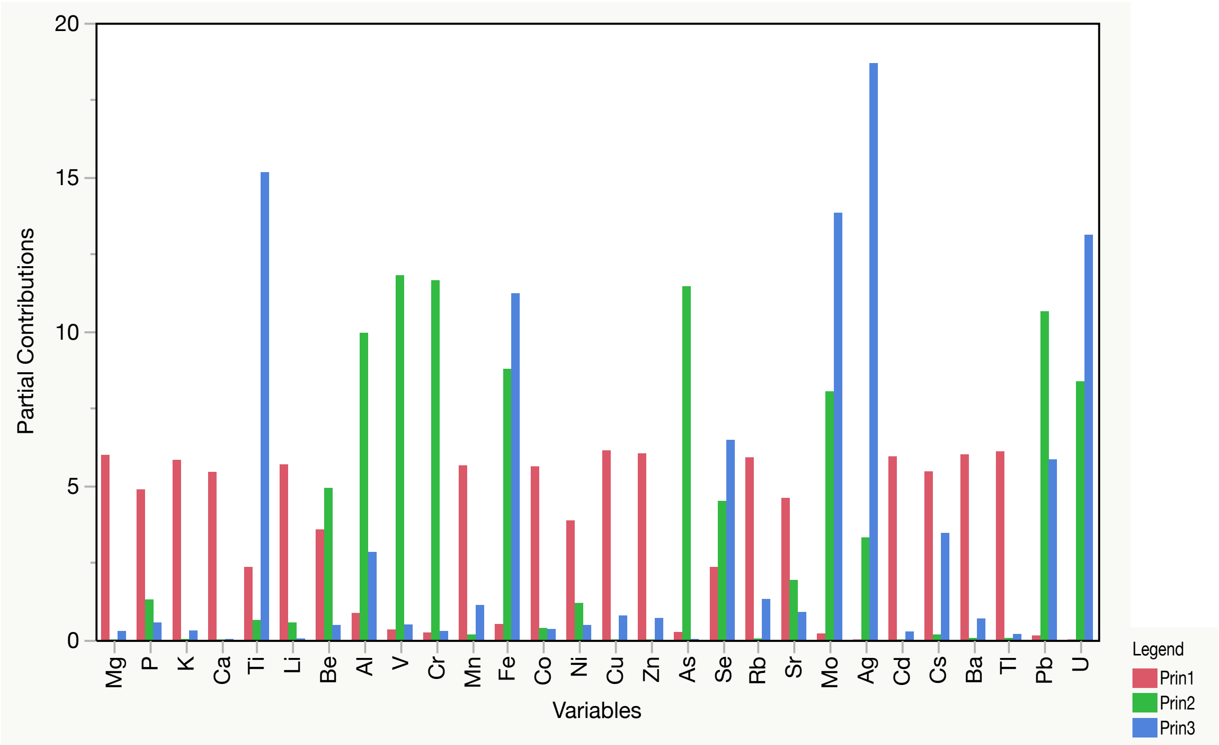

**Supplementary Figure 2. (A)** Mass of pyrogenic charcoal in ADE and Ultisol profiles extracted through acid-peroxide digestion. **(B)** Relative contribution of $C_4$ grass biomass to the total soil organic carbon calculated from the average stable carbon isotope ratios of ADEs and Ultisol profiles. Standard errors around the mean are shown in Figure 4. Regression lines show statistically significant trends with soil depth for ADE only (blue symbols).

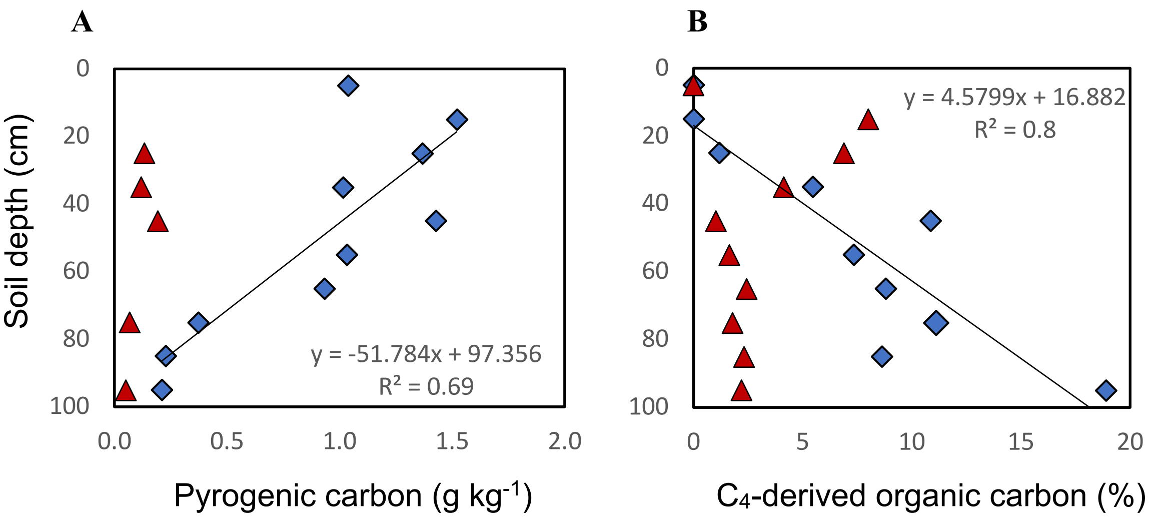

**Supplementary Figure 3.** Clay mineralogy characterization of a typical ADE and a typical Ultisol sample (40-50 cm depth) using quantitative X-ray diffraction (XRD) spectra following organic carbon removal and analysis of fine fraction. The spectra indicate similar mineralogy, which implies that differences in fertility were caused by exogenous input rather than local pedogenic processes. The analysis was performed using a Rigaku Ultima IV X-ray diffractometer (Tokyo, Japan) with a Cu tube at 40 kV and 40 mA at a scan rate of 2 degrees 2θ $min^{-1}$, with a Jade 9 (MDI, Livermore, CA) software for mineral identification of the clay fractions, in which gibbsite is identified by peaks at 4.85 Å and 4.38 Å and kaolinite is identified by a characteristic peak at 7.0 Å[1].

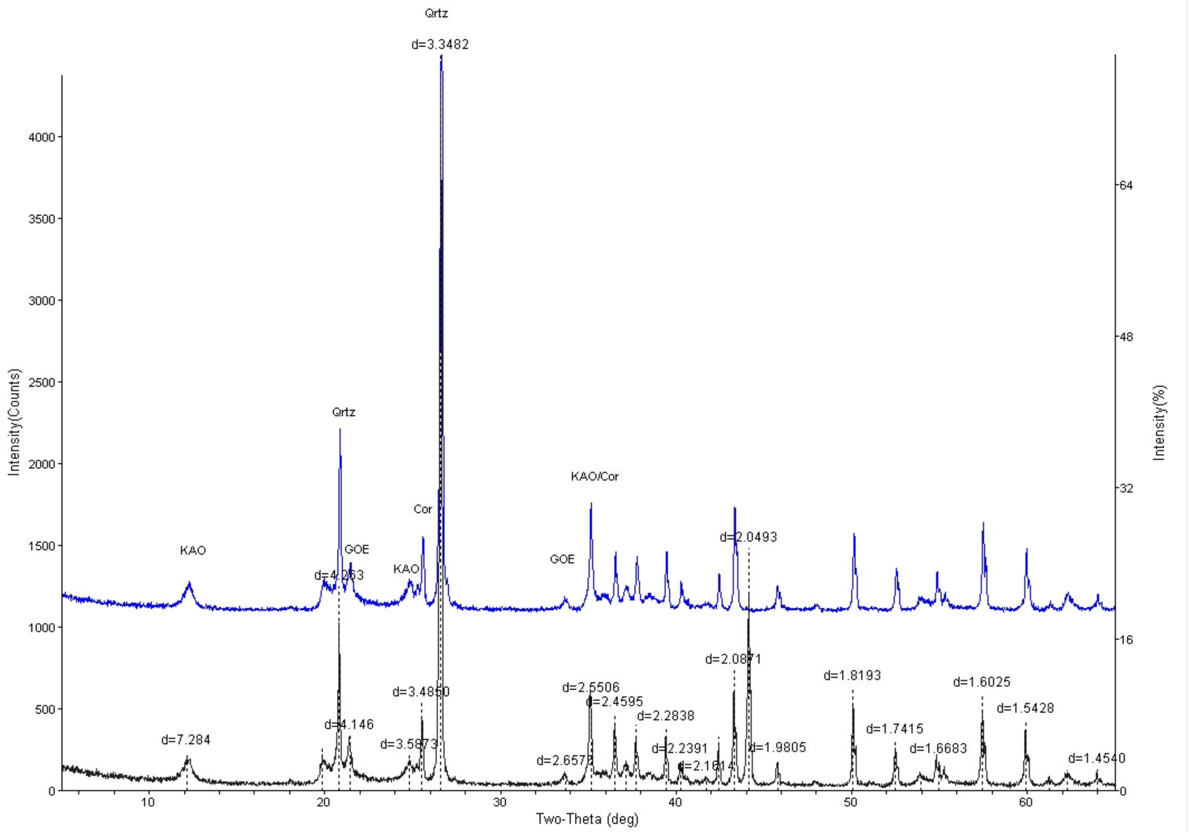

**Supplementary Figure 4**. **(A)** Isotopic ratios of strontium (Sr) as a function of soil depth and in relation to neodymium (Nd) isotope ratios. (**B)** Typical isotopic signatures of Sr and the radiogenic isotope of Nd εNd(0) - deviation from reference chondrite - across the Amazonian floodplain[2], which indicate exogenous alluvial inputs to ADE profiles but not to the adjacent Ultisol.

**A**

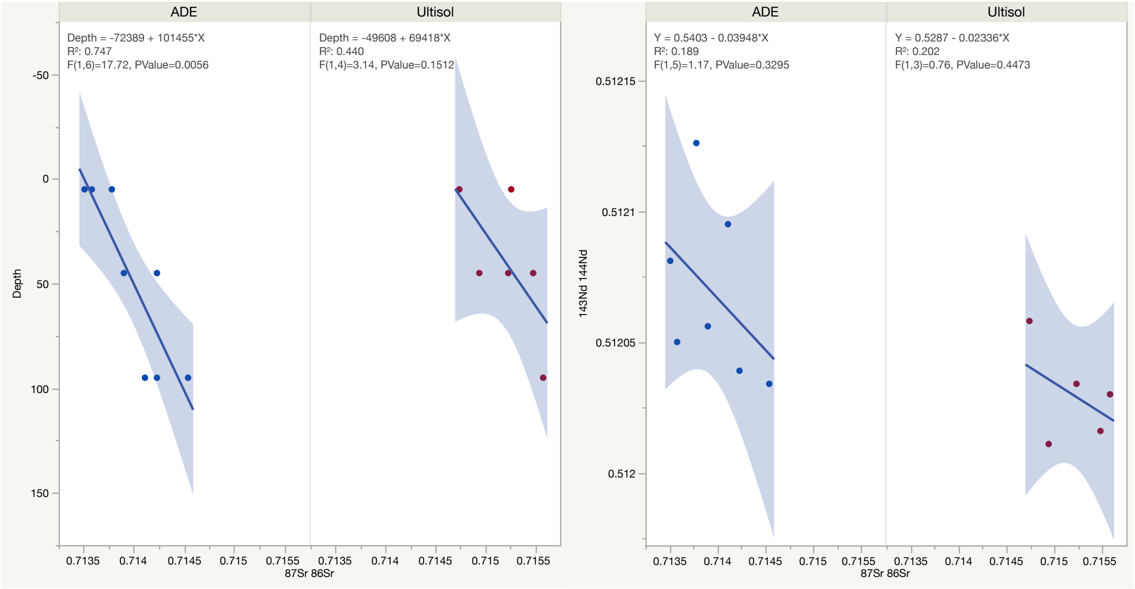


**B**

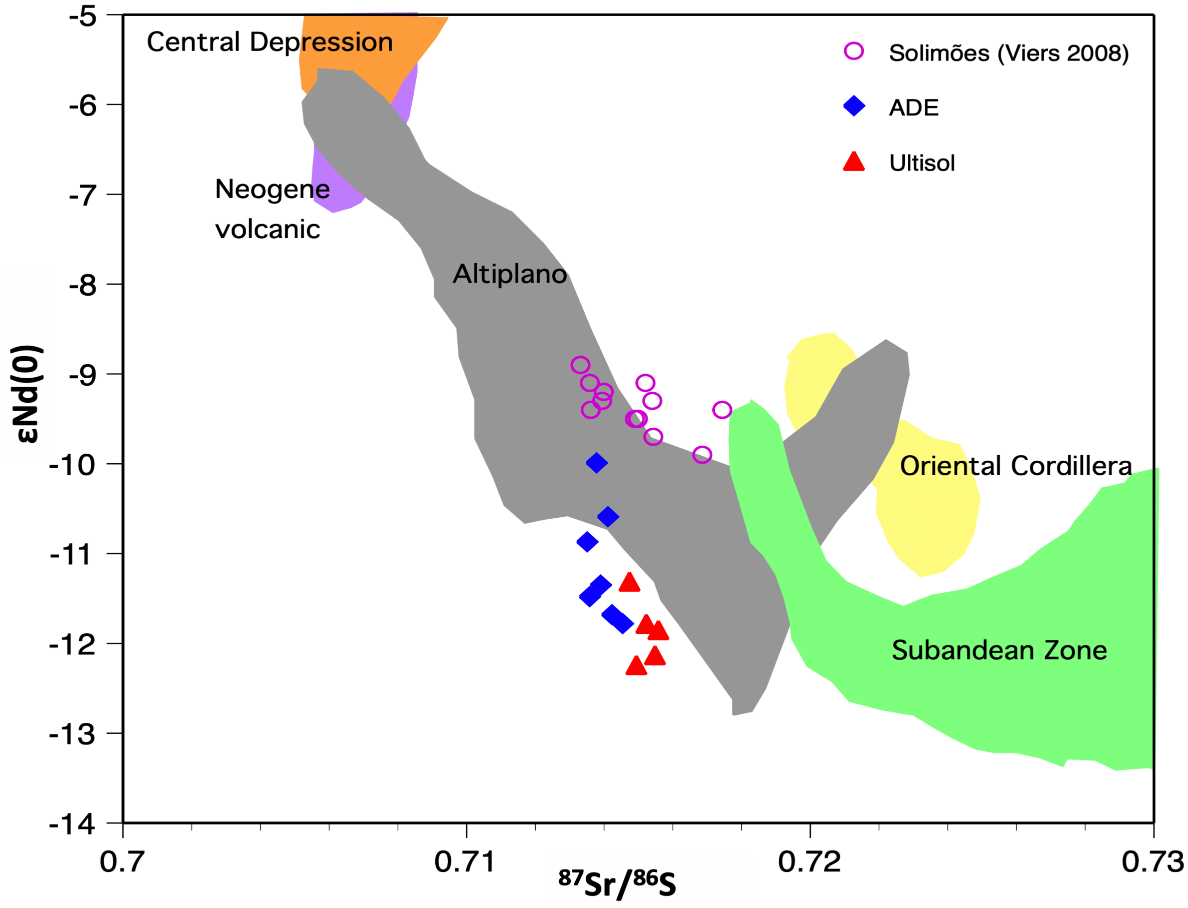

## Supplementary References


1. Aburto, F. A. & Southard, R. J. Thermal analysis mineral quantification and applications as a relative dating tool in moraine chronosequences. *Soil Sci. Soc. Am. J.* **80**, 502–515 (2016).
2. Roddaz, M. *et al.* Evidence for the control of the geochemistry of Amazonian floodplain sediments by stratification of suspended sediments in the Amazon. *Chem. Geol.* **387**, 101–110 (2014).
