# Supplementary material for: A new hypothesis for the origin of Amazonian Dark Earths
Source: Nat Commun. 2021 Jan 4;12:127. doi: 10.1038/s41467-020-20184-2 (PMC7782733; doi:10.1038/s41467-020-20184-2)
Supplement: Supplementary file 3 — Reporting Summary [file 41467_2020_20184_MOESM3_ESM.pdf]

nature research

Corresponding author(s): *Double-blind peer review submissions: write DBPR and your manuscript number here instead of author names.*

Last updated by author(s): *YYYY-MM-DD*

Reporting Summary

Nature Research wishes to improve the reproducibility of the work that we publish. This form provides structure for consistency and transparency in reporting. For further information on Nature Research policies, see our Editorial Policies and the Editorial Policy Checklist.

Statistics

For all statistical analyses, confirm that the following items are present in the figure legend, table legend, main text, or Methods section.

n/a	Confirmed	
☒	☐	The exact sample size (*n*) for each experimental group/condition, given as a discrete number and unit of measurement
☐	☒	A statement on whether measurements were taken from distinct samples or whether the same sample was measured repeatedly
☐	☒	The statistical test(s) used AND whether they are one- or two-sided<br>*Only common tests should be described solely by name; describe more complex techniques in the Methods section.*
☐	☒	A description of all covariates tested
☐	☒	A description of any assumptions or corrections, such as tests of normality and adjustment for multiple comparisons
☐	☒	A full description of the statistical parameters including central tendency (e.g. means) or other basic estimates (e.g. regression coefficient) AND variation (e.g. standard deviation) or associated estimates of uncertainty (e.g. confidence intervals)
☒	☐	For null hypothesis testing, the test statistic (e.g. *F*, *t*, *r*) with confidence intervals, effect sizes, degrees of freedom and *P* value noted<br>*Give P values as exact values whenever suitable.*
☒	☐	For Bayesian analysis, information on the choice of priors and Markov chain Monte Carlo settings
☒	☐	For hierarchical and complex designs, identification of the appropriate level for tests and full reporting of outcomes
☐	☒	Estimates of effect sizes (e.g. Cohen's *d*, Pearson's *r*), indicating how they were calculated

Our web collection on statistics for biologists contains articles on many of the points above.

Software and code

Policy information about availability of computer code

Data collection	n/a
Data analysis	All data analysis and visualizations were performed using open source software and code in R or SAS JMP

For manuscripts utilizing custom algorithms or software that are central to the research but not yet described in published literature, software must be made available to editors and reviewers. We strongly encourage code deposition in a community repository (e.g. GitHub). See the Nature Research guidelines for submitting code & software for further information.

Data

Policy information about availability of data

All manuscripts must include a data availability statement. This statement should provide the following information, where applicable:

- Accession codes, unique identifiers, or web links for publicly available datasets
- A list of figures that have associated raw data
- A description of any restrictions on data availability

The data used to produce all figures and supplementary materials present here are available at https://scholarsbank.uoregon.edu/

Field-specific reporting

Ecological, evolutionary & environmental sciences study design

All studies must disclose on these points even when the disclosure is negative.

Study description	We report new evidence from a well-studied ADE in the Brazilian Amazon, which compel us to reconsider its anthropic origin.
Research sample	Informed by anthropological studies, we sought to develop new evidence from one of the best studied ADE sites, which is thought to have originated from a large settlement less than 2000 years ago. The site is home to the Brazilian Agroforestry Research Station (EMBRAPA – CPAA), where the local ADE has been classified as a typical Anthrosol.
Sampling strategy	To improve understanding of ADE formation, we used EMBRAPA's reference profiles across a contiguous patch of ~12 hectares to select ~4.5 hectares for intensive sampling in a paired scheme that provides maximum contrast among ADE and adjacent Ultisols. All intensively sampled soils were under dense secondary evergreen rainforest (>12 m canopy height), which had not been managed for at least 40 years, since the research station was established. Across the study area, we measured mineral nutrient excess in ADE to estimate the input necessary to explain its origin. As a proxy for the overall amount of mineral nutrient input, we focused on phosphorus (P) and calcium (Ca) excess, which are two of the most limiting elements in tropical landscapes. We then quantified the association of P and Ca excess with the concentration of 26 other mineral elements, including strontium (Sr) and neodymium (Nd), whose isotopic signatures serve as proxies for mineral source. We then quantified total soil carbon, pyrogenic microcharcoal, and their respective radiocarbon (14C) dates for ADE and Ultisol profiles. Finally, considering the local anthropological context, we used carbon dates and nutrient excess data to estimate the chronology of land management and the population density needed to attain the observed gain in ADE fertility.
Data collection	A spatial grid was used to determine nutrient concentrations from reference soil profiles (Fig 1) was used to determine the sampling effort for the determination of total nutrients and stocks. A map of extractable concentration was used to guide the determination of total P and Ca stocks. Extractable P and Ca concentrations were 1 to 3 orders of magnitude greater within the ADE patch than in the surrounding Ultisol (Fig 2). The well-defined spatial pattern of extractable P and Ca from the most fertile ADE profiles (blue) to nutrient-depleted Ultisols (red) guided an in-depth characterization of elemental and isotopic composition performed along six transects ~100 m apart from one another, each containing 5 soil profiles, ~10 m apart from one another, for a total of 30 intensively sampled profiles (blue diamonds). Those profiles yielded samples for a maximum contrast comparison – that is, highest and lowest soil fertility along the site's southwest-northeast axis – on the basis of total nutrient stocks between ADEs and reference Ultisols (red triangles, Fig 3). To estimate total nutrient stocks, bulk density was multiplied by Ca and P concentrations at 10 cm depth increments up to 1 m in each profile (Fig 3 top panels). Without exception, all sampled profiles showed much larger nutrient stocks (expressed as megagrams per hectare; Mg ha-1) in the ADE than in the surrounding soil.
Timing and spatial scale	The EMBRAPA field station spans a contiguous patch of ~12 hectares to select ~4.5 hectares for intensive sampling in a paired scheme that provides maximum contrast among ADE and adjacent Ultisols. We systematically sampled the area using a gridded scheme and transects ~100 m apart from one another, each containing 5 soil profiles, ~10 m apart from one another, for a total of 30 intensively sampled profiles. We developed a soil age-depth model which span a chronology of nutrient and carbon inputs from ~7.5 thousand years ago to present day.
Data exclusions	n/a
Reproducibility	Hundreds of samples were used in a replicated hierarchical design use to compare ADEs and surrounding soil over time (thousands of years) and space (10s of thousands square meters).
Randomization	We used a systematic sampling scheme including grids and transects to capture the transition between the ADE patch and the surrounding soil.
Blinding	n/a

Did the study involve field work? ☒ Yes ☐ No

Field work, collection and transport

Field conditions	All intensively sampled soils were under dense secondary evergreen rainforest (>12 m canopy height), which had not been managed for at least 40 years, since the EMBRAPA research station was established.
Location	The EMBRAPA-CPAA site covers 210 hectares of managed and unmanaged land just north of the Solimões River near its confluence with the Negro River, within the sedimentary basin of the Amazon River. Satellite and field measurements show that the terrace is located ~40 m above the modern sea level, at a maximum of ~27 m above the modern river level, or ~10 m above the adjacent flood plain (3o 15' 6.8" S and 60º 13' 46.9" W).
Access & import/export	We used previously established access points and walked to each sampling locations causing little to no disturbance
Disturbance	Digging and sampling of soil profiles were performed without impacting the existing vegetation

Reporting for specific materials, systems and methods

We require information from authors about some types of materials, experimental systems and methods used in many studies. Here, indicate whether each material, system or method listed is relevant to your study. If you are not sure if a list item applies to your research, read the appropriate section before selecting a response.

Materials & experimental systems

n/a	Involved in the study
☒	☐ Antibodies
☒	☐ Eukaryotic cell lines
☒	☐ Palaeontology and archaeology
☒	☐ Animals and other organisms
☒	☐ Human research participants
☒	☐ Clinical data
☒	☐ Dual use research of concern

Methods

n/a	Involved in the study
☒	☐ ChIP-seq
☒	☐ Flow cytometry
☒	☐ MRI-based neuroimaging
